# Evaluation of locoregional invasiveness of small-sized non-small cell lung cancers by enhanced dual-energy computed tomography

**DOI:** 10.1186/s40644-016-0077-1

**Published:** 2016-07-26

**Authors:** Hironori Shimamoto, Shingo Iwano, Hiroyasu Umakoshi, Koji Kawaguchi, Shinji Naganawa

**Affiliations:** 1 Department of Radiology, Nagoya University Graduate School of Medicine, 65 Tsurumai-cho, Showa-ku, Nagoya, 466-8550 Japan; 2 Department of Thoracic Surgery, Nagoya University Graduate School of Medicine, 65 Tsurumai-cho, Showa-ku, Nagoya, 466-8550 Japan

**Keywords:** Contrast enhancement, Dual-energy CT, Functional imaging, Iodine quantification, Non-small cell lung cancer

## Abstract

**Background:**

To investigate the correlation between iodine-related attenuation of dual-energy computed tomography (DE-CT) and the histopathological invasiveness of surgically resected primary non-small cell lung cancers (NSCLCs) ≤ 3 cm in diameter.

**Methods:**

We selected 63 consecutive NSCLC lesions from 60 patients (32 males, 28 females; age range, 39–85 years; mean age, 68 years). After injection of iodinated contrast media, arterial phases were scanned using 140-kVp and 80-kVp tube voltages. Three-dimensional iodine-related attenuation (3D-IRA) of primary tumors at the arterial phase was computed using “lung nodule” application software. The corrected 3D-IRA normalized to the patient’s body weight and contrast medium concentration was then calculated. Single-factor analysis of variance (ANOVA) was used for comparison among tumor differentiation grade groups. Univariate and multivariate logistic regression analysis was used for the correlation between locoregional invasive tumor and clinical factors.

**Results:**

Resected tumors were histopathologically classified into well-differentiated (G1; *n* = 24), moderately-differentiated (G2; *n* = 28), and poorly-differentiated (G3; *n* = 11) groups by degree of tumor differentiation. The mean ± standard deviation of the 3D-IRA was 56.1 ± 22.6 HU in G1 tumours, 48.5 ± 23.9 HU in G2 tumours, and 28.4 ± 15.8 HU in G3 tumours; significant differences were observed between groups by ANOVA. (*p* = 0.005). Univariate logistic analysis showed that the 3D-IRA and corrected 3D-IRAs were significantly correlated with locoregional invasive tumors (*p* = 0.002 and *p* < 0.001, respectively). Multivariate logistic analysis revealed that only the corrected 3D-IRA was significantly correlated with tumor invasiveness (*p* = 0.003), while gender, clinical size, and solid/subsolid type were not (*p* = 0.950, *p* = 0.057 and *p* = 0.456, respectively).

**Conclusions:**

The 3D-IRA of small-sized NSCLCs was significantly associated with and invasiveness. Low 3D-IRA tumors tended to have greater invasiveness than high 3D-IRA tumors.

## Background

The prognosis and malignancy of primary lung cancer are affected by histopathological findings [1, 2]. For resectable non-small cell lung cancers (NSCLCs), preoperative diagnostic imaging is essential for determining the best therapeutic strategy, including the type of surgical procedure [3–5]. In the tumor, nodes, and metastasis (TNM) classification, T descriptors reflect tumor size and locoregional invasiveness. Tumor size can be measured definitively on thin-section computed tomography (CT) with a slice thickness of ≤ 1 mm. However, it is difficult to evaluate microscopic tumor invasiveness, such as pleural involvement, lymphatic permeation, and vascular invasion, even on thin-section CT.

Contrast-enhanced CT has been used in functional imaging to evaluate intratumoral necrosis, vascularity, and fibrosis, which are associated with invasiveness and prognosis [6–8]. A dual-energy CT (DE-CT) technique using two types of tube voltage has enabled quantification of the iodine-related attenuation of iodinated contrast material in the tumor after intravenous injection, without the need for an additional non-contrast CT scan [9–12]. It leads to radiation exposure reduction of a patient in a CT examination. Recently, Baxa et al. reported that iodine uptake at the arterial phase of dual phase contrast DE-CT is a feasible method in assessment of anti-EGFR therapy response for NSCLC [13]. Aoki et al. reported that iodine uptake at the arterial phase of contrast DE-CT is useful quantitative assessment for predicting lung cancer recurrence after stereotactic body radiotherapy [14]. That is, tumors with lower iodine uptake for pretreatment evaluation showed a worse prognosis after radiotherapy. These research findings indicate that iodine uptake at the arterial phase on contrast CT may be associated with some sort of tumor histopathology such as angiogenesis and hypoxic cell. Our recent study demonstrated that the three-dimensional iodine-related attenuation (3D-IRA; also known as “iodine volume”) of primary lung cancers measured by contrast-enhanced DE-CT was significantly associated with their differentiation grade [15]. Tumor grade correlates directly with microscopic tumor invasiveness [16–18]. Therefore, we speculated that the 3D-IRA might correlate indirectly with the microscopic invasiveness of small-sized NSCLCs. If that is true, a dual-energy technique would enable preoperative prediction of tumor invasiveness.

In this study, we reviewed preoperative data from contrast CT with dual-energy scanning as well as postoperative histopathological findings of NSCLCs ≤ 3 cm that were surgically resected, and validated the hypothesis that the 3D-IRA of the primary lesion could be a predictive factor for locoregional invasiveness.

## Methods

### Patient selection

We reviewed the medical records, post-operative pathological records, and preoperative DE-CT images of patients who underwent surgical lung resection for primary lung cancer at our hospital between April 2014 and March 2015. We limited these patients to those with NSCLCs of ≤ 3 cm in diameter that had been definitively diagnosed by pathological examination after surgical resection. Non-solid types of lung cancer that were invisible on mediastinal window settings for thoracic CT were excluded. Patients who received preoperative chemoradiotherapy were also excluded. We recorded patient characteristics (gender, age, and body weight) from medical records, and tumor characteristics (pathological tumor size, histological type, differentiation grade, lymphatic permeation, vascular invasion, and pleural involvement) from pathological records. Herein, the differentiation grade was classified into the following groups: G1, well-differentiated; G2, moderately-differentiated; and G3, poorly-differentiated. We defined locoregional invasive tumors as tumors that had at least one invasive pathologic finding (lymphatic permeation, vascular invasion, or pleural involvement) and non-invasive tumors as tumors that had no invasive pathologic findings [19, 20].

### CT scan protocol

All CT scans were performed with a dual-source CT scanner (SOMATOM Definition Flash; Siemens Healthcare, Tokyo, Japan) in the craniocaudal direction with inspiratory apnea. For vessel enhancement, 96 mL of non-ionic contrast medium (Proscope 300, 300 mgI/mL iopromide, Alfresa Pharma, Osaka, Japan; Optiray 320, 320 mgI/mL ioversol, Tyco Healthcare, Tokyo, Japan; or iopamiron 370, 370 mgI/mL iopamidol, Bayer Healthcare, Tokyo, Japan) were used with flow rates of 4.0 mL/sec. Iopromide was used for patients with body weights < 40 kg, ioversol was used for patients with body weights 40–59 kg, and Iopamidol was used for patients with body weights ≥ 60 kg. Injections were immediately followed by a saline chaser bolus of 20 mL at the same flow rate using a dual-barrel power injector (Dual Shot GX7; Nemoto Kyorindo, Tokyo, Japan).

In our institution, the dual-phase dynamic CTs were performed routinely to preoperative evaluation of lung cancer. After pre-contrast CT scan, the early phase of thoracic CT has been scanned for preoperative evaluation of pulmonary vessels by 3D-pulmonary angiography. The scan delay was evaluated by an automatic bolus tracking system with a circular ROI localized on the thoracic aorta. Scanning started automatically as the attenuation of the ROI increased 70 HU from baseline (about 20–25 sec. after start of injection). A dual-energy scan system (tube A at a peak voltage of 140 kVp and tube B at a peak voltage of 80 kVp) was used for both scans with collimation, 64 × 0.6 mm; gantry rotation speed, 285 msec. Additionally, the delayed phase of thoraco-abdominal CT has been scanned to evaluate lymph node metastasis and abdominal distant metastasis about 2 min later of contrast medium injection. Data were reconstructed with a slice thickness of 1.0-mm at 1.0-mm increments using iterative reconstruction techniques (SAFIRE, Siemens Healthcare, Tokyo, Japan).

### Analysis of dual-energy CT images

The 3D-IRA of the primary lesion was measured using the syngo “lung nodules” application accessory software [15, 21]. The DE-CT technique can create iodine-enhanced images from DE-CT raw data sets of 140kVp and 80kVp, and measure absolute quantification of iodine within the tumors. This application can automatically segment a pulmonary nodule and calculate the mean iodine related attenuation of pulmonary nodules with a three-material decomposition algorithm. A chest radiologist who had 21 years of experience reading thoracic CT scans segmented the primary tumor, measured the maximum diameter as clinical tumor size, and recorded the 3D-IRA.

Additionally, corrected 3D-IRA values to reduce the effect of body weight and iodine concentration of contrast medium, were calculated as follows:

$$ Corrected\kern0.5em  3D-IRA=\frac{raw\kern0.5em  data\kern0.5em  of 3D-IRA}{Concentration\kern0.5em  of\kern0.5em  contrast\kern0.5em  medium\kern0.5em \left[\mathrm{m}\mathrm{g}\mathrm{I}\right]/ Body\kern0.5em  weight\left[\mathrm{kg}\right]} $$

**Fig. 1:**
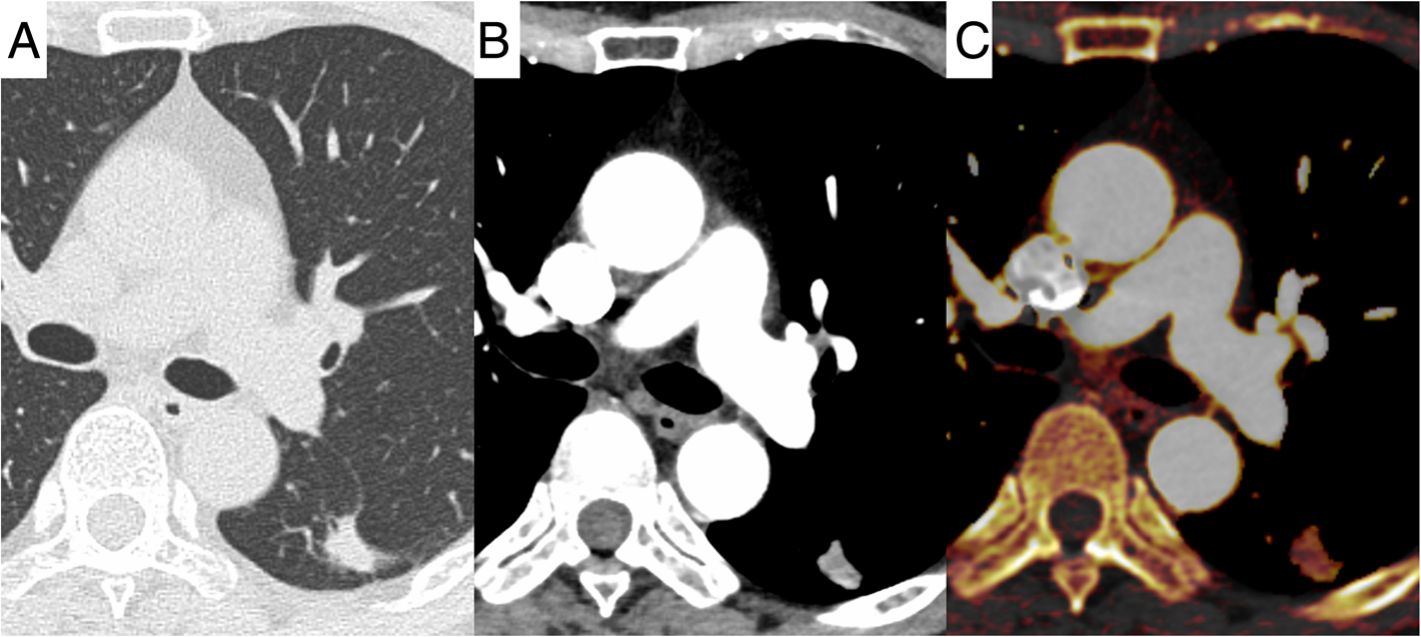
A 66-year-old female patient. A well-differentiated (G1) adenocarcinoma is observed in the left lower lobe. Lymphatic permeation, vascular invasion, and pleural involvement were all negative. **a** Lung window setting. **b** Mediastinal window setting. **c** Iodine-enhanced image of contrast-enhanced CT. The 3D-IRA is 47 HU, and the corrected 3D-IRA is 8.13

**Fig. 2:**
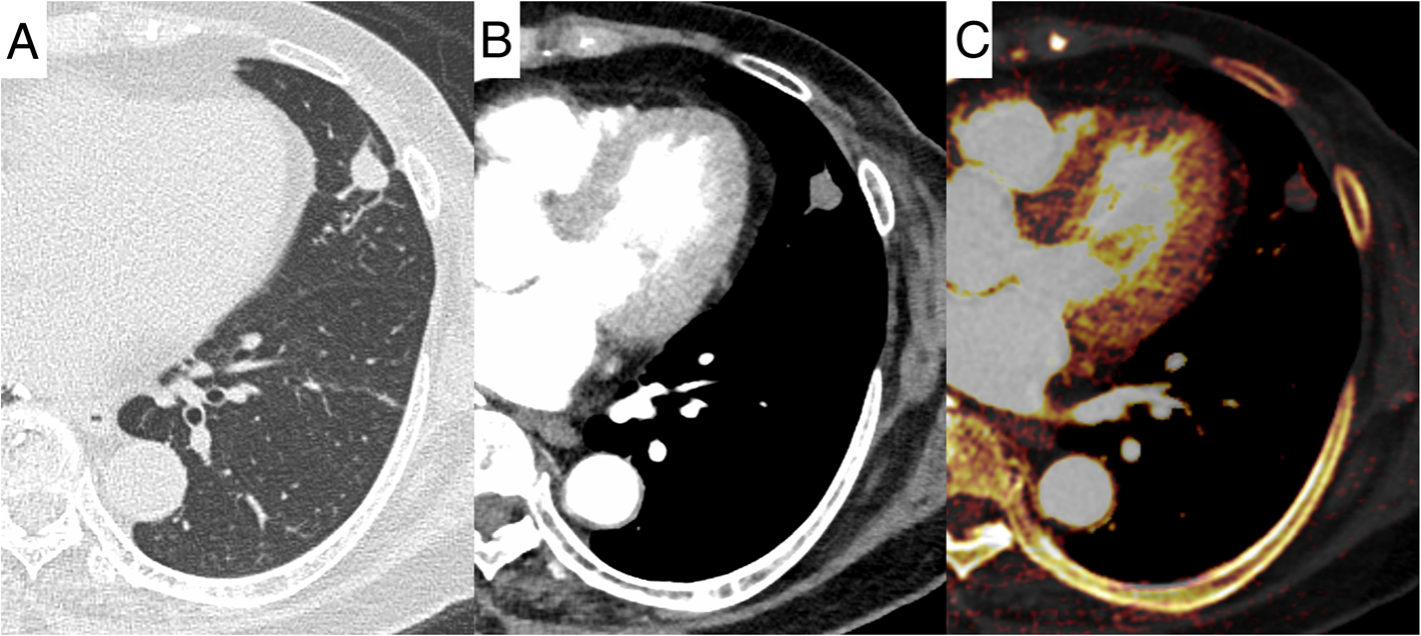
A 74-year-old female patient. A poorly-differentiated (G3) adenocarcinoma is observed in the left lingula. Vascular invasion, and pleural involvement were positive. **a** Lung window setting. **b** Mediastinal window setting. **c** Iodine-enhanced image of contrast-enhanced CT. The 3D-IRA is 16 HU, and the corrected 3D-IRA is 2.85

Representative cases of CT and iodine-enhanced images are shown in Figs. 1 and 2.

### Statistical analysis

First, among the three differentiation grade groups (G1 to G3), possible differences in patient characteristics were compared. Gender, ratio of part-solid nodules to solid nodules, locoregional invasive tumor, and lymph node metastases were compared by chi-square test. The age, body weight, clinical and pathological tumor size, 3D-IRA, and corrected 3D-IRA were compared by single-factor analysis of variance (ANOVA). Second, correlations between tumor characteristics and locoregional invasive tumors were individually evaluated using univariate logistic regression analysis. Third, areas under the curve of receiver operating characteristic (ROC) analysis for diagnosis of locoregional invasive tumor were compared between the 3D-IRAs and the corrected 3D-IRAs. Then, Youden’s index was used to determine a cut-off level that would indicate a locoregional invasive tumor. Last, multivariate logistic regression analysis was used for the correlation between locoregional invasive tumor and clinical factors for which the p-value was < 0.2 by univariate logistic analysis.

Analyses were performed using commercial statistical software: Excel 2010 (Microsoft Corp., Redmond, WA) and SPSS version 23 (IBM Corp., Amonk, NY). A p-value < 0.05 was considered statistically significant.

## Results

**Fig. 3:**
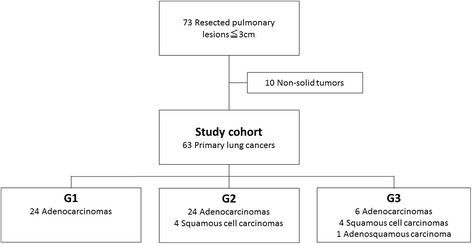
Study cohort flow chart. A total of 73 surgically resected pulmonary lesions were scanned preoperatively with DE-CT. After exclusion criteria were applied, 63 primary NSCLCs were included in the present analysis

A total of 73 resected pulmonary lesions were reviewed, and 10 lesions were excluded because they were non-solid type (Fig. 3). Finally, 63 consecutive primary lung cancer lesions from 60 patients were selected for analysis (32 males and 28 females; mean age, 67 years [range, 39–85 years]; mean body weight, 58.2 kg [range, 37–85 kg]). Of these lesions, 54 were adenocarcinomas, 8 were squamous cell carcinomas, and 1 was adenosquamous carcinoma.

Table 1 compares patient and tumor characteristics among differentiation grade groups. The mean ± standard deviation (SD) of 3D-IRA at the arterial phase was 56.1 ± 22.6 HU in G1 tumors, 48.5 ± 23.9 HU in G2 tumours, and 28.4 ± 15.8 HU in G3 tumours. The 3D-IRA was significantly different among the three differentiation grades (*p* = 0.005). The corrected 3D-IRA was also significantly different among the three differentiation grades (*p* = 0.005). Higher-grade tumors had lower 3D-IRA values. The percentage of locoregional invasiveness and lymph node metastases was significantly higher in high-grade tumors (*p* = 0.044 and *p* = 0.012, respectively). The ratios of female and part-solid type tumors were significantly higher in low-grade tumors (*p* = 0.001, and *p* <0.001, respectively). For age, body weight, and tumor size (both clinical and pathological), no significant differences were observed among the three groups (*p* = 0.912, *p* = 0.677, *p* = 0.758, and *p* = 0.069, respectively).

**Table 1:** Comparison of patient and tumor characteristics among differentiation grade groups

	G1	G2	G3	*p*-value
Male/female (n)	6/18	20/8	8/3	0.001
Age (years)	67 ± 7	67 ± 7	68 ± 12	0.912
Body weight (kg)	58.0 ± 13.8	57.3 ± 9.3	60.8 ± 9.3	0.677
Clinical size (mm)	22.4 ± 8.1	24.1 ± 8.2	23.3 ± 7.8	0.758
Pathological size (mm)	17.7 ± 6.9	21.2 ± 5.5	22.0 ± 6.7	0.069
Part-solid/Solid (n)	15/9	4/24	1/10	<0.001
3D-IRA (HU)	56.1 ± 22.6	48.5 ± 23.9	28.4 ± 15.8	0.005
Corrected 3D-IRA	9.05 ± 2.97	8.01 ± 4.09	4.81 ± 2.56	0.005
Locoregional invasive tumor (%)	25	54	64	0.044
Lymph node metastases (%)	0	25	36	0.012

3D-IRA, three-dimensional iodine related attenuation; cSize, clinical size of tumor on CT; pSize, pathological size of tumor

Table 2 shows the association between clinical or pathological factors and locoregional invasive tumor using univariate logistic regression analysis. Among the clinical factors, the 3D-IRA and the corrected 3D-IRA were significantly correlated with locoregional invasive tumors (*p* = 0.002 and *p* < 0.001, respectively). The gender, clinical tumor size, and internal opacity type of the clinical factors that were indicated as p-value <0.2 were adopted for subsequent multivariate logistic analysis. Among the pathological factors, the size and differentiation grade were significantly correlated with locoregional invasive tumors (*p* = 0.006 and *p* = 0.017, respectively).

**Table 2:** Univariate logistic analysis of clinical and pathological factors for locoregional invasiveness

	OR (95 % CI)	*p*-value
Clinical factors		
Male vs. Female	0.416 (0.150–1.155)	0.092
Body weight	1.008 (0.963–1.054)	0.741
Age	0.972 (0.914–1.034)	0.366
Clinical Size	1.061 (0.992–1.136)	0.085
Subsolid type vs. Solid type	2.683 (0.868–8.295)	0.087
3D-IRA	0.956 (0.930–0.983)	0.002
Corrected 3D-IRA	0.721 (0.595–0.873)	<0.001
Pathological factors		
Others as compared to adenocarcinoma	1.078 (0.261–4.456)	0.918
Pathological size	1.139 (1.038–1.251)	0.006
Differentiation grade	2.521 (1.177–5.398)	0.017

3D-IRA, three-dimensional iodine related attenuation; CI, confidence interval; OR, odds ratio; cSize, clinical size of tumor on CT; pSize, pathological size of tumor

**Fig. 4:**
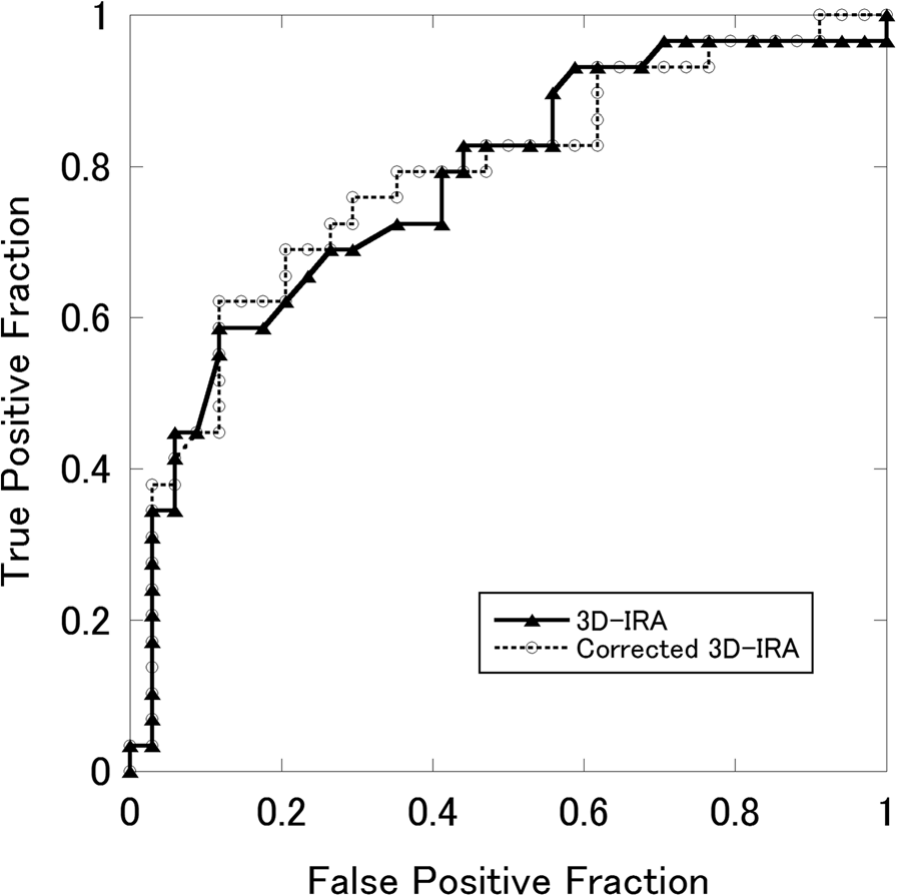
ROC curves of the 3D-IRA and the corrected 3D-IRA for diagnosis of locoregional invasive tumor

Figure 4 shows the ROC curves of the 3D-IRA and the corrected 3D-IRA for diagnosis of locoregional invasive tumors. The AUC of the 3D-IRA was 0.776 (95 % confidence interval, 0.658 - 0.894) and that of the corrected 3D-IRA was 0.781 (95 % confidence interval, 0.664–0.899). The AUC of the corrected 3D-IRA was higher than that of the 3D-IRA; however, the difference was not significant (*p* = 0.738). A suitable cutoff value for the corrected 3D-IRA was estimated to be 5.74. This value yielded 62.1 % sensitivity and 88.2 % specificity for determination of locoregional invasive tumors. From this result, the corrected 3D-IRA was used as a factor for multivariate logistic analysis.

Table 3 shows the correlation between locoregional invasiveness and four clinical factors, including gender, clinical tumor size, internal opacity, and corrected 3D-IRA by multivariate logistic regression analysis. Among these four factors, only the corrected 3D-IRA was significantly correlated with locoregional invasive tumors (*p* = 0.003). For gender, clinical tumor size, and solid vs. subsolid tumors, no significant differences were observed (*p* = 0.950, *p* = 0.057, and *p* = 0.456, respectively).

**Table 3:** Multivariate logistic analysis for locoregional invasive tumor

Factors	OR (95 % CI)	*p*-value
Male as compared to female	0.960 (0.267–3.456)	0.950
Clinical size	1.094 (0.997–1.200)	0.057
Subsolid as compared with solid	1.748 (0.402–7.598)	0.456
Corrected 3D-IRA	0.733 (0.596–0.901)	0.003

3D-IRA, three-dimensional iodine related attenuation; CI, confidence interval; OR, odds ratio; cSize, clinical size of tumor on CT

## Discussion

This study was designed to assess the role of enhanced DE-CT for small-sized NSCLC resected surgically and confirmed histopathologically. We researched the following two points: 1) Is the 3D-IRA associated with the differentiation grade, even in NSCLCs ≤ 3 cm in diameter? 2) Are the 3D-IRAs correlated with their invasiveness? This study revealed that the 3D-IRA was significantly correlated with differentiation grade. High-grade NSCLCs tended to show low contrast enhancement. In addition, only the corrected 3D-IRA was significantly correlated with locoregional invasiveness among four independent variables (gender, size, internal characteristics on CT, and corrected 3D-IRA) according to multivariate logistic regression analysis. Consequently, the present results demonstrated that contrast-enhanced DE-CT could diagnose malignant potential as well as fluorodeoxyglucose positron emission tomography/computed tomography (FDG-PET/CT).

The 3D-IRA and the corrected 3D-IRA of G3 NSCLCs were lower than those of G1 and G2 tumors. This result corresponds well with that of our previous study, which reported that G3 tumors showed significantly lower enhancement than G1 and G2 tumors [15]. This investigation suggested this occurred primarily due to intratumoral necrosis and fibrosis, which are closely correlated with invasiveness and prognosis of NSCLCs. In the present study, the frequency of locoregional invasive tumors in G3 tumors was higher than that in G1 and G2 tumors.

Therefore, we investigated the correlation between the 3D-IRA and degree of locoregional invasion in primary lesions. As we expected, according to univariate logistic regression analysis, the 3D-IRA, corrected 3D-IRA, pathological tumor size, and differentiation grade were significantly correlated with locoregional invasiveness. Furthermore, by multivariate logistic analysis, only the corrected 3D-IRA was significantly correlated with locoregional invasiveness among the clinical independent variables that were preoperatively obtainable. Thus, the 3D-IRA may be useful for selecting a therapeutic strategy, such as limited resection to preserve pulmonary function. Wedge resection or segmentectomy for small primary lung cancers are currently recommended for non-invasive tumors [22]. However, for small-sized lung tumors, it is sometimes difficult for transbronchial lung biopsy and CT-guided transcutaneous needle biopsy to determine the differentiation grade. An intraoperative diagnosis based on frozen sections and needle biopsies is also known to have limitations due to the potential risks of physical complications, false-negative results, and the implantation of cancer cells [23–25].

According to our univariate logistic regression analysis, both the 3D-IRA and corrected 3D-IRA that is the values were corrected by body weight and concentration of contrast materials, were significantly correlated with tumor invasiveness. Thus, we investigated which value was better correlated with invasiveness by ROC analysis. As a result, the corrected values showed a higher AUC than the raw ones, although no significant difference was observed. Therefore, we used the corrected values for multivariate logistic regression analysis.

PET/CT is useful for estimating the malignancy and invasiveness of lung tumors. The maximum standardized uptake value (SUVmax) has been commonly used to evaluate the invasiveness and prognosis of NSCLC. We believe that the 3D-IRA measured by DE-CT has three advantages over SUVmax on PET/CT for evaluation of lung cancer. First, 3D-IRA can be measured not only in large-sized but also small-sized tumors, as revealed in the present study. The lesion size is significantly associated with FDG uptake [26–28]. In particular, lesions ≤ 2 cm tended to have negative PET findings. Second, this was not influenced by respiratory variation because high-speed dual source CT can evaluate the whole lung in several seconds. Third, CT is less expensive than PET/CT. A previous study revealed that the SUVmax was negatively correlated with 3D-IRA [15]. Further investigation of the comparison with PET/CT is required.

This retrospective study has several limitations. First, we excluded non-solid type tumors from our study cohort, because it was difficult to measure 3D-IRA automatically using software. This represents a selection bias, as non-solid type lung adenocarcinoma is usually non-invasive [19, 29, 30]. Second, we could not evaluate differences between adenocarcinomas and squamous cell carcinomas because the number of squamous cell carcinoma cases was relatively small. Previous FDG-PET/CT studies demonstrate that SUVs of squamous cell carcinomas are higher than those of adenocarcinomas [20, 31, 32]. Third, since the number of patients with lymph node metastasis was relatively small, we could not evaluate the correlation with the 3D-IRA of small-sized NSCLCs. Finally, the radiation dose on contrast enhanced CT was not compared between DE-CT and conventional single energy CT. However, the recent study of Uhrig et al. demonstrated that abdominal DE-CT is feasible without increasing radiation dose or deteriorating image quality, even compared to single energy CT [33].

## Conclusions

The 3D-IRA of small-sized NSCLCs measured by DE-CT was significantly associated with differentiation grade and invasiveness. Primary lung cancers with lower 3D-IRA tended to have a high grade and be more invasive.

## Abbreviations

3D-IRA, three-dimensional iodine-related attenuation; ANOVA, analysis of variance; AUC, area under the curve; DE-CT, dual-energy computed tomography; FDG-PET/CT, fluorodeoxyglucose positron emission tomography/computed tomography; NSCLC, non-small cell lung cancer; ROC, receiver operating characteristic; ROI, region of interest

## References

[1] Chung CK, Zaino R, Stryker JA, O’Neill M, DeMuth WE (1982). Carcinoma of the lung: evaluation of histological grade and factors influencing prognosis. Ann Thorac Surg.

[2] Kozu Y, Maniwa T, Takahashi S, Isaka M, Ohde Y, Nakajima T (2013). Risk factors for both recurrence and survival in patients with pathological stage I non-small-cell lung cancer. Eur J Cardiothorac Surg.

[3] Kishimoto M, Iwano S, Ito S, Kato K, Ito R, Naganawa S (2014). Prognostic evaluations of small size lung cancers by 18 F-FDG PET/CT and thin-section CT. Lung Cancer.

[4] Iwano S, Yokoi K, Taniguchi T, Kawaguchi K, Fukui T, Naganawa S (2013). Planning of segmentectomy using three-dimensional computed tomography angiography with a virtual safety margin: Technique and initial experience. Lung Cancer.

[5] Billé A, Pelosi E, Skanjeti A, Arena V, Errico L, Borasio P (2009). Preoperative intrathoracic lymph node staging in patients with non-small-cell lung cancer: accuracy of integrated positron emission tomography and computed tomography. Eur J Cardiothorac Surg.

[6] Iwano S, Koike W, Matsuo K, Okada T, Shimoyama Y, Naganawa S (2011). Correlation between dual-phase dynamic multi-detector CT findings and fibrosis within lung adenocarcinoma tumors. Eur J Radiol.

[7] Spira D, Neumeister H, Spira SM, Hetzel J, Spengler W, von Weyhern CH (2013). Assessment of tumor vascularity in lung cancer using volume perfusion CT (VPCT) with histopathologic comparison: a further step toward an individualized tumor characterization. J Comput Assist Tomogr.

[8] Wang Y, Wang J-a, Liang K-r, Liang M-z, X-g L (2014). Correlations between tumor stroma characters and dynamic enhanced MDCT findings in nodular pulmonary adenocarcinoma. J Comput Assist Tomogr.

[9] Zhang LJ, Yang GF, Wu SY, Xu J, Lu GM, Schoepf UJ (2013). Dual-energy CT imaging of thoracic malignancies. Cancer Imaging.

[10] Schmid-Bindert G, Henzler T, Chu TQ, Meyer M, Nance JW, Schoepf UJ (2012). Functional imaging of lung cancer using dual energy CT: how does iodine related attenuation correlate with standardized uptake value of 18FDG-PET-CT?. Eur Radiol.

[11] Baxa J, Vondrakova A, Matouskova T, Ruzickova O, Schmidt B, Flohr T (2014). Dual-phase dual-energy CT in patients with lung cancer: assessment of the additional value of iodine quantification in lymph node therapy response. Eur Radiol.

[12] Sudarski S, Hagelstein C, Weis M, Schoenberg SO, Apfaltrer P (2015). Dual-energy snap-shot perfusion CT in suspect pulmonary nodules and masses and for lung cancer staging. Euro J Radiol.

[13] Baxa J, Matouskova T, Krakorova G, Schmidt B, Flohr T, Sedlmair M, Bejcek J, Ferda J. Dual-Phase Dual-Energy CT in Patients Treated with Erlotinib for Advanced Non-Small Cell Lung Cancer: Possible Benefits of Iodine Quantification in Response Assessment. Eur Radiol. 2015. doi:10.1007/s00330-015-4092-6. 10.1007/s00330-015-4092-6 26563350

[14] Aoki M, Hirose K, Sato M, Akimoto H, Kawaguchi H, Hatayama Y, Fujioka I, Tanaka M, Ono S, Takai Y. Prognostic impact of average iodine density assessed by dual-energy spectral imaging for predicting lung tumor recurrence after stereotactic body radiotherapy. J Radiat Res. 2016. doi:10.1093/jrr/rrv100. 10.1093/jrr/rrv100 PMC4973636 26826198

[15] Iwano S, Ito R, Umakoshi H, Ito S, Naganawa S (2015). Evaluation of lung cancer by enhanced dual-energy CT: association between three-dimensional iodine concentration and tumour differentiation. Br J Radiol.

[16] Al-Alao BS, Gately K, Nicholson S, McGovern E, Young VK, O’Byrne KJ (2014). Prognostic impact of vascular and lymphovascular invasion in early lung cancer. Asian Cardiovasc Thorac Ann.

[17] Pechet TT, Carr SR, Collins JE, Cohn HE, Farber JL (2004). Arterial invasion predicts early mortality in stage I non-small cell lung cancer. Ann Thorac Surg.

[18] Poncelet AJ, Cornet J, Coulon C, Collard P, Noirhomme P, Weynand B (2008). Intra-tumoral vascular or perineural invasion as prognostic factors for long-term survival in early stage non-small cell lung carcinoma. Eur J Cardiothorac Surg.

[19] Iwano S, Kishimoto M, Ito S, Kato K, Ito R, Naganawa S (2014). Prediction of pathologic prognostic factors in patients with lung adenocarcinomas: comparison of thin-section computed tomography and positron emission tomography/computed tomography. Cancer Imaging.

[20] Ito R, Iwano S, Kishimoto M, Ito S, Kato K, Naganawa S (2015). Correlation between FDG-PET/CT findings and solid type non-small cell cancer prognostic factors: are there differences between adenocarcinoma and squamous cell carcinoma?. Ann Nucl Med.

[21] Chae EJ, Song JW, Krauss B, Song KS, Lee CW, Lee HJ, Seo JB (2010). Dual-energy computed tomography characterization of solitary pulmonary nodules. J Thorac Imaging.

[22] Yoshida J, Ishii G, Hishida T, Aokage K, Tsuboi M, Ito H (2015). Limited resection trial for pulmonary ground-glass opacity nodules: case selection based on high-resolution computed tomography-interim results. Jpn J Clin Oncol.

[23] Ozeki N, Iwano S, Taniguchi T, Kawaguchi K, Fukui T, Ishiguro F (2014). Therapeutic surgery without a definitive diagnosis can be an option in selected patients with suspected lung cancer. Interact Cardiovasc Thorac Surg.

[24] Gould MK, Donington J, Lynch WR, Mazzone PJ, Midthun DE, Naidrich DP (2013). Evaluation of individuals with pulmonary nodules: when is it lung cancer? Diagnosis and management of lung cancer, 3rd ed: American College of Chest Physicians evidence-based clinical practice guidelines. Chest.

[25] Wiener RS, Schwartz LM, Woloshin S, Welch HG (2011). Population-based risk for complications after transthoracic needle lung biopsy of a pulmonary nodule: an analysis of discharge records. Ann Intern Med.

[26] Iwano S, Ito S, Tsuchiya K, Kato K, Naganawa S (2013). What causes false-negative PET findings for solid-type lung cancer?. Lung Cancer.

[27] Suzawa N, Ito M, Qiao S, Uchida K, Takao M, Yamada T (2011). Assessment of factors influencing FDG uptake in non-small cell lung cancer on PET/CT by investigating histological differences in expression of glucose transporters 1 and 3 and tumour size. Lung Cancer.

[28] Nomori H, Watanabe K, Ohtsuka T, Naruke T, Suemasu K, Uno K (2004). Evaluation of F-18 fluorodeoxyglucose (FDG) PET scanning for pulmonary nodules less than 3 cm in diameter, with special reference to the CT images. Lung Cancer.

[29] Asamura H, Hishida T, Suzuki K, Koike T, Nakamura K, Kusumoto M (2013). Radiographically determined noninvasive adenocarcinoma of the lung: survival outcomes of Japan Clinical Oncology Group 0201. J Thorac Cardiovasc Surg.

[30] Ohde Y, Nagai K, Yoshida J, Nishimura M, Takahashi K, Suzuki K (2003). The proportion of consolidation to ground-glass opacity on high resolution CT is a good predictor for distinguishing the population of non-invasive peripheral adenocarcinoma. Lung Cancer.

[31] Muto J, Hida Y, Kaga K, Ohtaka K, Okamoto S, Tamaki N (2014). Use of maximum standardized uptake value on fluorodeoxyglucose positron-emission tomography in predicting lymph node involvement in patients with primary non-small cell lung cancer. Anticancer Res.

[32] Li M, Sun Y, Liu Y, Han A, Zhao S, Ma L (2010). Relationship between primary lesion FDG uptake and clinical stage at PET-CT for non-small cell lung cancer patients: An observation. Lung Cancer.

[33] Uhrig M, Simons D, Kachelrieß M, Pisana F, Kuchenbecker S, Schlemmer HP (2016). Advanced abdominal imaging with dual energy CT is feasible without increasing radiation dose. Cancer Imaging.

